# Next‐Generation Genotoxicology: Using Modern Sequencing Technologies to Assess Somatic Mutagenesis and Cancer Risk

**DOI:** 10.1002/em.22342

**Published:** 2019-11-11

**Authors:** Jesse J. Salk, Scott R. Kennedy

**Affiliations:** ^1^ Department of Medicine, Division of Medical Oncology University of Washington School of Medicine Seattle Washington; ^2^ TwinStrand Biosciences Seattle Washington; ^3^ Department of Pathology University of Washington Seattle Washington

**Keywords:** chemical carcinogenesis, cancer risk assessment, *in vivo* mutation, error‐corrected NGS, consensus sequencing, single‐cell sequencing, single molecule sequencing

## Abstract

Mutations have a profound effect on human health, particularly through an increased risk of carcinogenesis and genetic disease. The strong correlation between mutagenesis and carcinogenesis has been a driving force behind genotoxicity research for more than 50 years. The stochastic and infrequent nature of mutagenesis makes it challenging to observe and to study. Indeed, decades have been spent developing increasingly sophisticated assays and methods to study these low‐frequency genetic errors, in hopes of better predicting which chemicals may be carcinogens, understanding their mode of action, and informing guidelines to prevent undue human exposure. While effective, widely used genetic selection‐based technologies have a number of limitations that have hampered major advancements in the field of genotoxicity. Emerging new tools, in the form of enhanced next‐generation sequencing platforms and methods, are changing this paradigm. In this review, we discuss rapidly evolving sequencing tools and technologies, such as error‐corrected sequencing and single cell analysis, which we anticipate will fundamentally reshape the field. In addition, we consider a variety emerging applications for these new technologies, including the detection of DNA adducts, inference of mutational processes based on genomic site and local sequence contexts, and evaluation of genome engineering fidelity, as well as other cutting‐edge challenges for the next 50 years of environmental and molecular mutagenesis research. Environ. Mol. Mutagen. 61:135–151, 2020. © 2019 The Authors. *Environmental and Molecular Mutagenesis* published by Wiley Periodicals, Inc. on behalf of Environmental Mutagen Society.

## INTRODUCTION

**Figure 1:**
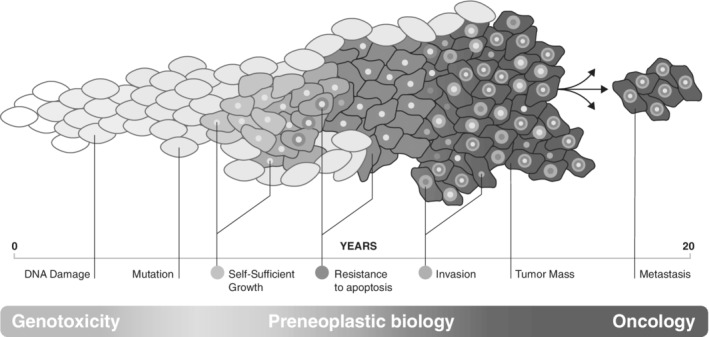
The genesis of cancer. Cancer exists on a continuum. Mutations arise as a result of repair and replication errors due to endogenous processes and environmental factors. These mutations are the substrate for neoplastic clonal evolution: those that confer a proliferative or survival advantage upon the host cell will be naturally selected. Carcinogens promote tumorigenesis by increasing the rate of mutation or by enhancing net‐positive selection. Given the often impractically long lag‐time between a carcinogenic insult and overt tumor formation, technologies that are able to sensitively detect DNA damage, mutation induction, and clonal outgrowths are essential tools in a genetic toxicologist's armamentarium.

Exposure to environmental factors has been known to alter the genetic makeup of organisms since the seminal work by Hermann Muller in 1927 showing that *Drosophila* exposed to X‐rays led to new heritable traits (Muller 1927). Other environmental factors, including ultraviolet light and reactive chemicals, were reported soon after (Stadler and Sprague 1936; Auerbach et al. 1947). It wasn't until the publication of the structure of DNA in 1953, and the subsequent description of DNA polymerases that a mechanism linking environmental exposures to mutagenesis and heritable changes became fully apparent (Watson and Crick 1953; Bessman et al. 1958; Lehman et al. 1958). The ensuing years led to a rapid expansion of studies to catalog and better understand environmental mutagens. By the mid‐1970's, experiments in rodent models indicated that the majority of known mutagens were, in fact, carcinogenic (McCann et al. 1975). Because of the strong link, as well as the desire to save both time and money, evaluating the mutagenic potential of a compound has become a *de facto* surrogate for carcinogenicity (Fig. 1). A detailed treatment of the regulatory aspects of this important subject area is provided elsewhere in this issue (Heflich et al. 2019).

A number of key technologies have been developed over the past 50 years to quantify genotoxicity in both *in vitro* and *in vivo* settings. The spontaneous mutation rate in normal somatic mammalian cells is estimated to be in the range of 10^−8^–10^−9^ mutations per nucleotide per cell division (Lynch 2010). Directly detecting these rare events at the DNA sequence level is technically challenging (Milholland et al. 2017)—the molecular equivalent of “Where's Waldo?” (Handford 2007). Not only does one need to screen a very large number of nucleotides cells to obtain a reasonable statistical confidence of mutant frequencies, but the method for detecting mutations must also have an error rate below the true mutant frequency.

To circumvent these challenges, most standard mutagenesis assays rely on some means of biological enrichment, whereby mutations are detected by a selectable phenotype they create. While the specifics differ, the general approach relies on exposing bacterial or mammalian cells to a putative mutagen and then quantifying the ratio of cells harboring a mutation in a selectable marker to the number of cells present in the absence of selection. *In vitro* selection‐based mutagenesis assays include the classic Ames assay and several mammalian cell culture‐based mutation tests, such as *HPRT* and *APRT* (Ames et al. 1973; Thompson et al. 1980). While highly effective, *in vitro* assays have several limitations that make them imperfect surrogates for human toxicology, including differences in metabolic activation/inactivation of the tested compound, the use of only a small number of cell types, and continuous cellular proliferation that can result in potential “jackpot” events. *In vivo* assays include transgenic rodent models, such as the MutaMouse and the BigBlue mouse/rat assays which involve multistep transfer of DNA from mutagen‐exposed rodents into phage and then into bacteria (Kohler et al. 1991; Myhr 1991). By taking advantage of the *in vivo* context, transgenic animals solve some of the issues inherent to the *in vitro* assays. As a testament to their utility, these selection based assays are still widely used decades after their initial development. A history detailing the importance of these technologies is provided in this issue by DeMarini (DeMarini 2019).

While these methods are ubiquitous in both research and regulatory settings, reliance on selection to quantify mutagenesis comes at a cost. The nuclear genome is a dynamic system with spatially heterogeneous levels of biomolecular activity, such as transcription, chromatin accessibility, adjacent nucleotide context, and DNA repair which strongly modulate susceptibility to mutagenesis across the genome (Hodgkinson and Eyre‐Walker 2011). Most such assays rely on a single reporter locus that is often artificially introduced. Furthermore, the number of possible mutations that render a selectable phenotype may be limited in some cases, leading to an underestimation bias arising from the inability to observe variants that result in no phenotypic changes (eg, synonymous mutations). Lastly, selectable markers are not always portable between different experimental systems and are currently limited to a few common organismal models.

Technologies that directly identify mutations in DNA of primary tissue samples without necessitating a multistep selection and cloning process would open up opportunities to identify mutagenic compounds in a more unbiased manner. One such method is the *Pig‐a* assay (Bryce et al. 2008). This assay uses flow cytometry to rapidly screen millions of cells for those that lack expression of a particular nonessential surface protein due to inactivating mutations. Helpfully, this approach can be applied to both humans and model organisms, but generally only to red blood cells, limiting its applicability to the other tissues in the body and making it difficult to confirm the exact nature of the mutations themselves (mature red blood cells are enucleate).

Several sensitive biochemical assays for mutation detection have been developed, often based on resistance to endonuclease cleavage or allele‐specific PCR. While extremely sensitive, these methods are either too low‐throughput or excessively narrow in scope (ie, interrogate only one or a few bases) to gain wide usage (Parsons and Heflich 1997; Bielas and Loeb 2005). Thus, until the advent of modern next‐generation sequencing (NGS), also referred to as massively parallel sequencing, selection‐based assays have been the dominant technology for evaluating mutagenesis.

**Figure 2:**
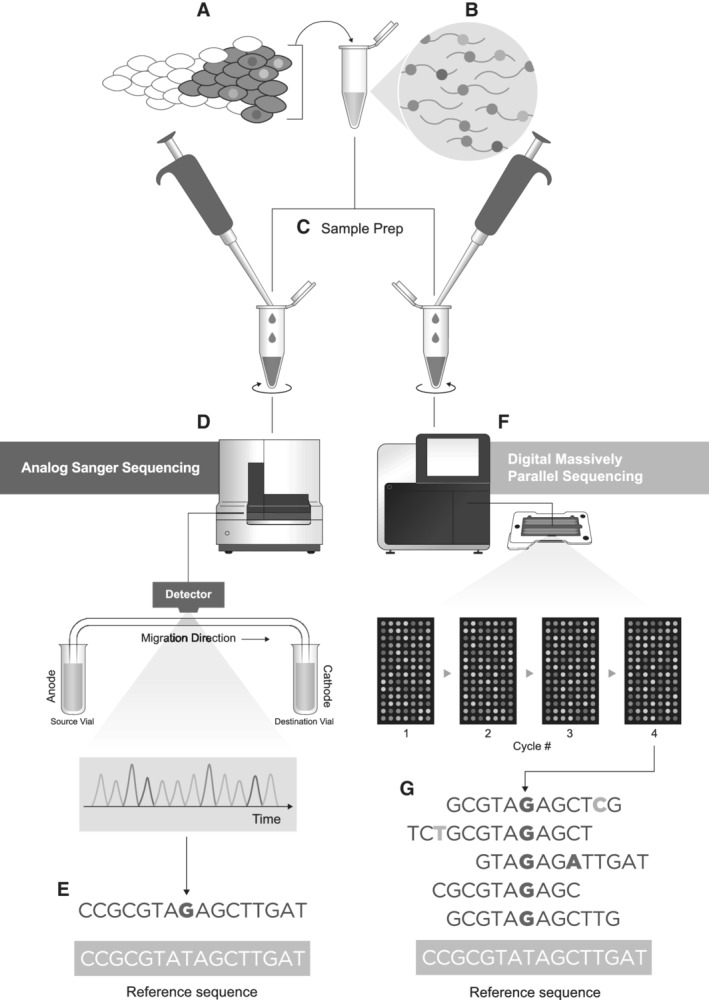
Analog *vs*. digital DNA sequencing. A common need in genetic toxicology is to identify mutations in cell populations. The appropriateness of the sequencing technology depends on mutational clonality. **(A)** Clonal mutations are those present in all or most cells in a tissue (gray), whereas subclonal mutations (colors) are present in only a subset. **(B)** When DNA is extracted from a tissue, a mutation's clonality is reflected in the isolated molecules that are then **(C)** prepared for sequencing. **(D)** With traditional Sanger sequencing, all molecules from the same genomic region are genotyped together *en masse* in a capillary system, which produces an analog output (electropherogram tracing) that is the average of many different DNA molecules. **(E)** Generally only substantially clonal mutations can be reliably detected. **(F)** In contrast, next‐generation sequencing operates by massively parallel sequencing of millions of individual molecules digitally. On the widely used Illumina sequencing‐by‐synthesis platform, this is accomplished by flowing fluorescently labeled nucleotides across a surface coated with small biochemically generated colonies of individual molecules (clusters), and recording the sequence of colors of each cluster through multiple cycles of addition. **(G)** The resulting output is not a single sequence, but millions of individual ones that reflect both clonal and subclonal mutations down to approximately 1% abundance.

Beginning in approximately 2005, NGS has revolutionized many of fields of life science, including cancer biology, population genetics, evolutionary biology, and cellular biology. There are a several commercially available NGS platforms that differ in their underlying approaches to obtaining sequence information, but all share the ability to simultaneously obtain this information from tens of thousands to billions of individual DNA templates. Consequently, it is now possible to obtain data on a genome‐wide scale. In addition, NGS technologies are read‐based. This “digital tabulation” approach differs from conventional Sanger sequencing methods by obtaining the nucleotide sequence of many individual DNA molecules, thus enhancing the ability to detect minor mutant populations within a heterogeneous DNA mixture which is generally the context in which somatic mutagenesis occurs (Metzker 2010; Fig. 2).

The distinct advantages offered by NGS will revolutionize environmental mutagenesis and toxicology by overcoming past limitations and providing new opportunities for study. Despite its transformative potential, NGS has only recently gained attention in this field, as several key technical hurdles have now been overcome. In this review, we discuss the advances in modern DNA sequencing technologies that are enhancing the ability to detect low‐frequency mutagenic events and DNA damage. We review cutting edge applications that are currently being facilitated by these new technologies and others we see on the horizon.

## NEXT‐GENERATION SEQUENCING TECHNOLOGIES

In genetic toxicology, most applications of NGS to date have focused on augmenting and enhancing the throughput of well‐established genotoxicity assays—for example, increasing the throughput of sequencing of mutant shuttle vectors or plaques from transgenic models (Yuan et al. 2011; Besaratinia et al. 2012; Beal et al. 2015; Chang et al. 2015). Other applications have included non‐mutational assessments of genetic toxicology, such as epigenetic and transcriptional changes, induced by chemical exposure (Chauhan et al. 2016; Li et al. 2017a), as well as the whole‐genome detection of environmentally induced *de novo* mutations in offspring of exposed individuals (Reviewed in [Marchetti et al. 2019; Godschalk et al. 2019]).

However, neither of these cases fully realize the aspirational goal of being able to directly measure genotoxin‐induced DNA mutations in any tissue type of any organism. This is because modern sequencing platforms are not without their limitations. Given the random nature of genotoxic insults, genetic toxicology assessment in the absence of biological selection generally necessitates being able to detect low‐frequency somatic mutations in a large population of non‐mutant DNA molecules. In theory, DNA subpopulations of any size should be detectable by NGS when assessing a sufficient number of molecules. However, while notably better than Sanger sequencing, standard NGS platforms still generate errors at a substantial rate. Mistakes arising during DNA preparation, amplification, cluster generation, and the many steps of sequencing itself typically result in ~1% artifactual bases, and this background can be significantly higher in certain sequence contexts (reviewed in (Salk et al. 2018)). In contrast, the biological mutation frequency of even heavily mutagenized animals is on the order of one mutation per million nucleotides. Therefore, to detect chemically induced somatic mutations, far more sensitive NGS technologies are needed.

### Error‐Corrected Next‐Generation Sequencing

Several approaches have been employed to improve the accuracy of NGS. Initial efforts to reduce the technical error rate of NGS focused on bioinformatic filtering of low‐confidence sequences. For example, a number of variant calling tools filter the data based on the distribution of variants with the sequencing reads or require variants to be seen in multiple independent sequencing reads in both read orientations (Wang et al. 2013). More recently, statistical approaches have been specifically developed to improve variant calling by modeling the error profile of specific sequencing platforms (Wei et al. 2011; Wilm et al. 2012). These bioinformatic approaches allow for the detection of variants to mutant fraction of ~0.5%. This level of sensitivity is effective for clonally expanded mutations (such as those arising in the germ line or found in tumors) but is still orders of magnitude above the spontaneous mutant frequency of DNA (Martincorena et al. 2015, 2017).

In addition to bioinformatic filtering, enzymatic removal of DNA damage has been shown to reduce the number of false variant calls in NGS. For example, 8‐oxo‐dG and cytidine deamination, two of the most common DNA damaging events, can be biochemically removed with the damage‐specific glycosylases FPG and UDG, respectively. Combinations of glycosylases with other repair enzymes can further repair damage‐induced artifacts (Chen et al. 2017b), yet not all mutagenic lesions are recognized by these enzymes, nor is the fidelity of *in vitro* repair perfect, and the possibility exists that these approaches introduce new errors at low levels.

**Figure 3:**
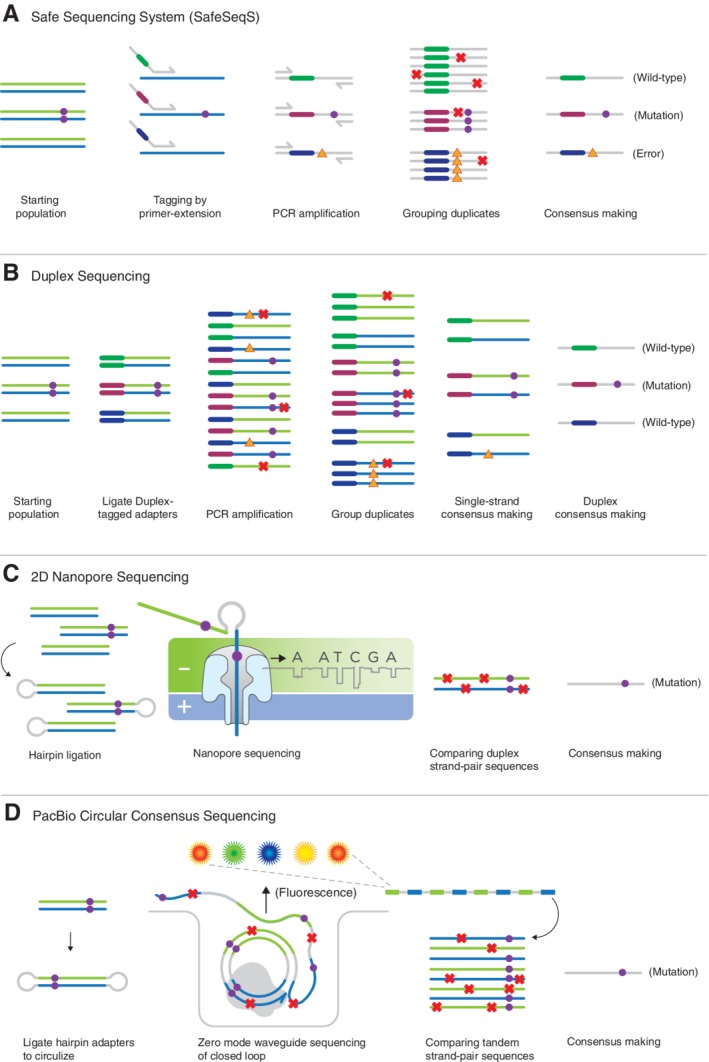
Techniques for error corrected DNA sequencing (ecNGS). The highest accuracy NGS methods rely on sequencing‐by‐consensus, whereby data from multiple sequence reads derived from an original molecule are combined to reduce the impact of sequencing or sample preparation errors in each read. **(A)** The SafeSeqS approach uses random molecular barcodes applied to PCR primers to uniquely tag PCR amplicons, which are then further amplified and sequenced. Variation within the sequence of reads with identical tags can be discounted as technical artifacts (X's). Some errors that occur during the first extension cycle may escape correction (triangles). **(B)** Duplex Sequencing relies on ligation to apply molecular barcodes to both strands of original double‐stranded molecules. These are used alone or in combination with fragmentation points to uniquely label both strands such that derivative sequence reads from each strand can be directly related back to their founder strand and compared to those from its complement. The method is significantly more accurate that single‐stranded consensus‐making methods but is more sequencing‐intensive. **(C)** 2D sequencing on nanopore platforms uses physical linkage of the two strands of an original duplex, which are then sequenced together without the need for amplification. The method is fast and simple, but nanopore platforms are lower accuracy and throughput than more widely used sequencing‐by‐synthesis platforms. **(D)** Circular Consensus Sequencing on the PacBio single‐molecule platform similarly links the two strands of an original double‐stranded with hairpins to allow multiple sequencing passes across both original strands. As with 2D, lower raw platform accuracy and throughput are drawbacks but very long reads can be obtained.

The approach to error‐corrected next‐generation sequencing (ecNGS) that has, thus far, proven the most significant for improving accuracy is consensus‐based error correction (Fig. 3). The technique relies on the general concept of grouping reads that are copies derived from an original DNA molecule and then bioinformatically creating a consensus sequence from the related molecules. An important aspect of this approach is the need to identify related reads, which can be accomplished by the use of a uniquely identifying “molecular barcode” (also referred to as “unique molecular identifier” (UMI), “single molecule identifier”, or simply a “tag”) for each original DNA fragment that will be propagated to all daughter molecules during amplification and sequencing. Molecular barcodes can be comprised of unique fragmentation shear points, exogenously introduced degenerate DNA sequences, or a combination of the two. Importantly, they must provide enough sequence diversity to minimize the probability that two independent molecules will share the same molecular barcode by chance.

Several groups introduced the idea of using molecular barcodes to correct sequencing‐based errors, but these initial studies focused on non‐variant detection applications, such as read assembly and molecular counting (Hiatt et al. 2010; Casbon et al. 2011; Fu et al. 2011). With the publication of the SafeSeqS method, Kinde et al. definitively introduced the idea of using molecular barcoding for improving the accuracy of mutation detection by applying single‐stranded molecular barcodes in the tails of PCR primers, reducing the error rate to ~10^−5^ (Kinde et al. 2011; Fig. 3A). A number of variations on this concept have been published, including single‐molecule molecular inversion probes (Hiatt et al. 2013), circular sequencing (Lou et al. 2013), and CypherSeq (Gregory et al. 2016), among others. Consensus‐making techniques that label just one strand of original double‐stranded molecules or cannot distinguish the identity of the two strands markedly reduce sequencer‐based artifacts, such as base calling errors and amplification errors introduced during cluster generation, thereby reducing the methodological background by two to three orders of magnitude and making it possible to confidently identify rare variants at ~0.1% abundance (Salk et al. 2018).

However, methods relying on single‐stranded tagging are fundamentally limited by base selectivity of DNA polymerases which, at best, have error rates of ~10^−6^ (McInerney et al. 2014). Of particular relevance is the elevated rate of misincorporations at sites of mutagenic DNA damage. For example, the presence of 8‐oxo‐dG adducts or deaminated cytosine bases (dU) dramatically increases the misincorporation rate of polymerases upon traversal of the lesion (Shibutani et al. 1991; Lindahl 1993). These misincorporation events can be propagated to daughter molecules during PCR, making it difficult to distinguish between artifacts induced by chemical adducts and *bona fide* variants occurring at dC and dG bases. Moreover, different DNA adducts are repaired with vastly different efficiencies by the cell (Wood 1996). Thus, with these methods, experiments involving mutagen exposure run the risk of detecting the presence of both adducts and true mutations. Given that mammalian cells are quite adept at recognizing and repairing adducts *in vivo*, it is incorrect to equate adducts with mutations (the vast majority will be repaired before mutation occurs *in vivo*). Cumulatively, these factors contribute to a practical detection limit of ~10^−4^–10^−5^, depending on DNA quality and experimental conditions (reviewed in (Salk et al. 2018)). This is excellent for many applications but does not reach the accuracy threshold needed for direct mutagenesis assessment.

Some mutagenic compounds are capable of increasing the mutation frequency of DNA by ~1000‐fold or more. However, because the spontaneous mutation frequency of the mammalian nuclear genome is normally very low (on the order of one‐per‐10‐million base pairs), even a 1000‐fold increase is still below what is reliably detectable by single‐strand UMI‐based methods. Extending the concept of molecular barcoding to include asymmetric double‐stranded UMIs allows for the sequencing information derived from complementary strands of original double‐stranded to be compared for an additional level of error correction. Double‐stranded consensus calling requires uniquely identifying each original DNA molecule (ie, a unique molecular identifier) and its constituent strands (ie, a strand‐defining element) in a way that allows the sequences to be related to each other. Duplex Sequencing was the first method to use double‐stranded consensuses to remove both sequencer and early PCR derived errors (Schmitt et al. 2012; Kennedy et al. 2014; Fig. 3B). A number of derivative approaches, including BiSeqS (Mattox et al. 2017), muSeq (Kumar et al. 2018), and BotSeqS (Hoang et al. 2016), have been developed that establish molecular barcodes and strand‐defining elements *via* partial bisulfite treatments or random shear points in conjunction with ultra‐low genome coverage. With all these approaches, the theoretical error rate of double‐strand consensus methods is estimated to be ~10^−9^, which roughly reflects the square of the error rate for single‐strand molecular barcoding methods. Duplex methods have been used by a number of groups to study the occurrence of mutations arising from a number of genotoxic species, including smoking, aflatoxin, aristolochic acid, urethane, benzo[a]pyrene, and reactive oxygen species (Kennedy et al. 2013; Hoang et al. 2016; Chawanthayatham et al. 2017).

### Single‐Cell Sequencing Technologies

Typical NGS protocols rely on fragmenting the genomes of thousands of cells. The result is a mixture of contributing cellular genotypes when the underlying population is heterogeneous. In such situations, ecNGS approaches are needed to detect these rare variants in the sea of wild‐type sequences if their abundance is below approximately 1%. However, the creation of a heterogeneous mixture of DNA fragments from many different genomes eliminates the ability to identify variants to within the same cell, potentially underestimating the mutagenic potential of a compound that may only bio‐accumulate in certain cell types (or cell division states). Sequencing the DNA from single cells overcomes this problem and ensures that observed mutations came from the same cell.

Typical single‐cell sequencing (SCS) protocols require isolation of individual cells followed by lysis and usually some form of whole‐genome amplification to generate enough DNA for sequencing (Zong et al. 2012; Fu et al. 2015; Dong et al. 2017; Chen et al. 2017a). Somatic mutations would typically be heterozygous (absent recombination events or loss of heterozygosity) and expected to be present in 50% of reads mapping to the genomic position of interest. SCS methods have been able to successfully detect structural variants (Wang et al. 2012), copy‐number variations (Navin et al. 2011), and single nucleotide variants (Dong et al. 2017) on a genome wide scale. To date, SCS approaches have not been widely deployed to evaluate genotoxicity at the single‐cell level. However, recent work by the Vijg group demonstrated the ability of SCS to detect mutations induced by mutagenic exposure with N‐ethyl‐N‐nitrosourea, indicating its potential utility (Dong et al. 2017).

Another barrier to deploying SCS for genotoxicity applications is throughput and, by extension, cost. In response to the need for more high‐throughput methods, microfluidic sorting of cells (Rinke et al. 2014), nano‐well technologies (Gierahn et al. 2017), and emulsion droplet partitioning technologies (Klein et al. 2015) have been developed and have increased throughput up to ~10,000 cells. A promising new approach to massively parallel SCS, termed combinatorial cellular indexing, uses intact fixed cells or nuclei as “reaction vessels” to physically partition the nucleic acids of interest. A unique combination of DNA sequences (ie, a cellular index) are enzymatically introduced to all the nucleic acids present within each cell/nucleus, a technique sometimes referred to as “combinatorial indexing” or “split‐pool barcoding.” Because all sequencing reads derived from nucleic acids from the same cell share the same cell‐specific index, the sequencing data can be computationally grouped and assigned to a specific cell. This approach offers the ability to examine hundreds of thousands of cells without the need for complex single‐cell handling equipment and has been used to study structural variations, transcriptomics, and epigenetics (Cusanovich et al. 2015; Cao et al. 2017; Vitak et al. 2017; Rosenberg et al. 2018). The steady improvements in throughput and cost makes SCS increasingly attractive for answering important hypotheses about genotoxicity that can only be answered at the level of individual cells. The efficient combination of SCS with high‐accuracy single‐molecule consensus sequencing methods would be an extremely powerful tool of the future.

### Direct Single‐Molecule Sequencing

Several mutagenesis assays are routinely used to detect clastogenic compounds, such as the micronucleus and chromosomal aberration assays (Araldi et al. 2015). Although effective from a risk assessment perspective, these classic tools do not yield specific sequence information. Modern sequencing platforms are able to detect structural variants, but with the added benefit of providing detailed sequence information and genomic location. While Illumina's reversible terminator dye technology, with its reasonably good accuracy and high throughput, is well suited to detect single‐nucleotide changes, it is currently limited to read lengths of less than 300 bases (600 bases for paired‐end). Short read length significantly hinders the ability to detect large structural variations and genomic rearrangements. Therefore, structural variants are bioinformatically detected by searching for reads spanning a break point or inferred by read‐pairs mapping farther apart than a few kilobases or to different chromosomes (Alkan et al. 2011). Bioinformatic detection tends to have highly variable sensitivity and specificity rates due to the size of the structural variant, occurrence of chimeric PCR products prior to sequencing, overlapping clusters or read‐hopping on the sequencer, or the occurrence of erroneous read mapping arising from pseudogene sequences elsewhere in the genome (Alkan et al. 2011; Kosugi et al. 2019).

Direct single‐molecule sequencing (SMS) is a relatively new technology that offers a number of advantages over short read sequencing methods. Two different SMS technologies are currently commercially available: single‐molecule real‐time sequencing (SMRT; commercialized by Pacific Biosciences) and nanopore (commercialized by Oxford Nanopore Technologies). van Dijk et al. (2018) provides a detailed comparison of these two technologies. Both approaches produce very long reads (10–250 kb) and directly sequence genomic DNA without the need for intermediate PCR amplification. The elimination of PCR chimeras and the addition of more sequence information within a single read significantly reduce mis‐mappings and increases the probability of spanning breakpoints, minimizing false positives.

Although these technologies enhance the ability to detect structural variants, they exhibit much higher error rates in the detection of single nucleotide variants, often as high as 15%–20% (Quail et al. 2012; Ross et al. 2013; Jain et al. 2017). However, these platforms are amenable to platform specific variations of consensus sequencing to reduce their high false‐positive rates. For example, in SMRT‐based platforms, circularized original DNA molecules can be sequenced repeatedly with a highly processive DNA polymerase and a “circular consensus sequence” made for each template, improving the accuracy of SNV calls by several orders of magnitude (Travers et al. 2010; Fig. 3C). Nanopore‐based technologies, however, are not yet amendable to significant consensus error correction by repeated sequencing of the same molecule. Currently, a type of double‐strand consensus can be made by affixing a hairpin adapter to the DNA fragments such that the two strands can be sequentially sequenced in a reverse complementary fashion, referred to as “two‐directional” sequencing (Fig. 3D). This approach has been reported to reduce the error rate to ~3%–5% (Jain et al. 2015; Tyler et al. 2018). Two recent methods, termed Rolling‐Circle to Concatameric Consensus and Intramolecular‐ligated Nanopore Consensus Sequencing, offer the possibility of increasing the accuracy of nanopore‐based platforms by implementing a circular consensus sequencing‐like approach, analogous to what is performed on the PacBio platform (Li et al. 2016; Volden et al. 2018).

## NEXT‐GENERATION SEQUENCING APPLICATIONS

Modern sequencing platforms are rapidly transforming the ability to detect, quantify, and characterize genomic DNA at an ever increasing rate and scale. These technologies open up new potential avenues of research that are likely to have a profound impact on the study of genomic toxicology and mutagenesis. We highlight a number of emerging applications for modern sequencing platforms that are of high relevance for genotoxicity studies.

### Adduct Detection by Sequencing

Genotoxic compounds that induce mutagenesis typically do so by chemical modification of the DNA that induces base mis‐insertion by DNA polymerases during genome replication or repair. The majority of damage is effectively removed by multifaceted cellular repair processes before mutation occurs (Sancar et al. 2004). However, the level of DNA damage and efficiency of repair can vary widely by genomic context and damage type, with some adducts and genomic locations being essentially unrepaired (Chang et al. 2015; Perera et al. 2016; Geacintov and Broyde 2017). As such, there is far from a one‐to‐one relationship between the presence of an adduct and risk of mutagenesis. Indeed, this is the impetus behind the widely used comet assay that grossly quantifies the aggregate presence of DNA break and adducts but has the limitation of not providing sequence context or genomic location information. While outside the scope of this review, the presence of unrepaired DNA adducts has been shown to lead to increases in transcriptional mutagenesis and significant physiological consequences, even when the underlying DNA sequences is unchanged (reviewed in (Brégeon and Doetsch 2011)).

A number of approaches have been developed to take advantage of modern sequencing platforms to assess the distribution of DNA adducts on a genome wide scale and, frequently, at single‐nucleotide resolution. Current short‐read technologies, such as the Illumina platform, are typically unable to directly detect DNA adducts, so the presence of chemical alterations must be inferred by other means. One strategy is the detection of read start or termination positions. This approach relies on the ability of bulky lesions, such as alkyl groups, to block the DNA polymerases during the PCR steps used in library preparation (Hu et al. 2016; Hu et al. 2017; Wu et al. 2018). The result is that the DNA fragments being sequenced will terminate immediately adjacent to the blocking moiety. The use of DNA repair enzymes or chemical treatments has also been employed to specifically cleave DNA at sites of damage followed by adapter ligation and sequencing. The result is similar to the above, whereby the 5′‐end of a read denotes a site immediately adjacent to a site of damage. This strategy has been used to detect UV (Mao et al. 2016; Hu et al. 2017), cisplatin (Hu et al. 2016), and bulky alkyl adducts (Mao et al. 2017; Aloisi et al. 2019). The presence and location of ribose bases in DNA can be similarly inferred, simply by inducing breaks with alkaline hydrolysis (Orebaugh et al. 2018).

Another frequently used strategy to infer DNA damage employs enrichment for, or depletion of, DNA fragments containing adducts. Depletion‐based approaches make use of enzymatic removal of adducts that render those DNA fragments unsequenceable. The readout is a drop in coverage areas of the genome prone to DNA damage relative to undamaged ones (Bryan et al. 2014). This approach exhibits poor sensitivity when adducts are present in only a small minority of DNA molecules, as is the case in many *in vivo* applications. One solution is to enrich adduct‐containing molecules *via* immunoprecipitation of DNA bearing specific adducts or bound repair proteins (ie, base excision repair or nucleotide excision repair, *etc*.) (Bryan et al. 2014; Hu et al. 2017, Hu et al. 2016; Li et al. 2017b). In an analogous approach, base adducts that are poorly targeted by immunoprecipitation can be chemically modified to make them amendable for capture (Wu et al. 2018). Both methods can significantly improve detection of damage or repair activity on a genome‐wide scale.

An advantage of many single‐molecule sequencing platforms is that many DNA adducts can be directly detected without prior manipulation. In the case of the PacBio SMRT sequencing technology, chemical modifications to the template base affect the kinetics of dNTP incorporation by DNA polymerases in a defined way that is relatively specific to each adduct (Clark et al. 2011). Most studies have focused on endogenous epigenetic modifications (ie, methylation), but the methods and statistical analysis employed by these studies could easily be adapted to genotoxicity applications.

Challenges in detecting blocking lesions are one notable limitation for this polymerase‐based approach. Nanopore technologies, on the other hand, are well suited for identifying bulky adducts. Base‐calling is accomplished by observing changes in ionic current/impendence that are specific to the template base as it passes through the nanopore structure (reviewed in (Deamer et al. 2016)). Base modifications are detectable because they alter this characteristic profile in an adduct‐specific way. Most efforts have focused on detecting endogenous methylations (Laszlo et al. 2013; Schreiber et al. 2013), but an increasing number of reports are beginning to characterize a wider variety of exogenous DNA adducts more relevant to genetic toxicology, including pyrimidine dimers, benzo[a]pyrine, 8‐oxo‐dG, abasic sites, and double‐strand cross‐links (An et al. 2012; Wolna et al. 2013; An et al. 2015; Perera et al. 2015; Zhang et al. 2015).

### Characterizing Genotoxicity by Mutational Signatures

One of the primary goals of genotoxicity testing is to link specific exposures to mutagenesis and, ultimately, carcinogenesis. Controlled exposure studies in animal models are currently the gold standard for relating exposure to carcinogenicity. However, the linking of mutagenic exposure to cancer in human populations is far more complex and largely depends on population level epidemiological studies (Wild 2008). With some rare exceptions, such as skin cancer with sun exposure and cervical cancer with human papillomavirus, definitive attribution of a specific instance of cancer to a specific genotoxic event is extremely difficult, especially when compounded with the naturally occurring accumulation of mutations in cancer relevant genes during aging (reviewed in (Risques and Kennedy 2018)). Tools that enable detection of genotoxic exposure in humans, and more closely link its relationship to cancer, would have a profound impact on clinical medicine and public health, as well as important legal and ethical implications.

**Figure 4:**
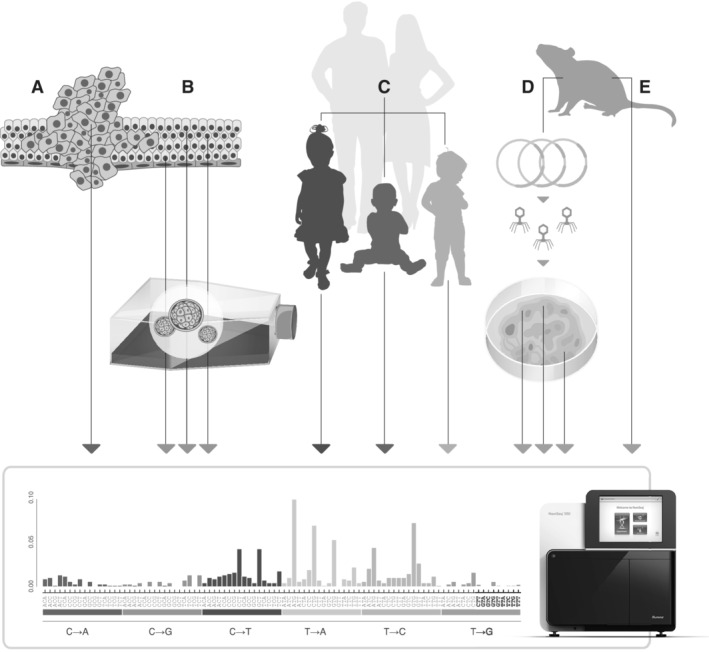
Approaches for assessing mutational signatures. Mutational spectra, particularly polynucleotide mutational signatures, provide important mechanistic insights into mutational processes. Most of what we know about these patterns has come from natural or artificial means of single cell cloning. **(A)** Exome or whole‐genome sequencing of tumor populations reflects the somatic processes operative in the founding cell of the most recent clonal sweep. **(B)** Single cells can be cloned from cultured populations exposed to known or suspected mutagens to assess their mutational signatures **(C)** The clonal variants present in individuals that were not present in their parents reflects the state of mutational processes during gametogenesis or early embryogenesis. **(D)** Sequencing of cloned cells or molecules from certain selection‐based mutagenicity assays can be used similarly, although the patterns may be distorted by the selection system itself. **(E)** With ecNGS, it is now possible to obtain mutational spectra by directly sequencing DNA from any tissue of any organism.

The relative incidence of different types (or spectra) of single‐base substitutions are nonrandom and strongly depends on the specific nature of the mutagen. On their own, simple mutation spectra (ie, A→G *vs*. C→A) have limited specificity due to significant overlap between different mutagens and their predominant mutation type. Local sequence context, however, strongly influences the frequency of a given type of mutation. The identity of flanking nucleotides adds a great deal of additional information that can be harnessed to better indicate the exact etiology of observed mutations (Fig. 4).

Data generated by The Cancer Genome Atlas and other large‐scale sequencing efforts have provided an opportunity to identify many distinct mutational patterns in a wide variety of cancer types. By taking into account known cancer biology and patient medical history, analysis of the tumor mutation patterns can, in some cases, provide a correlative link between exposure and the observed mutational patterns; for example, high levels of mutations seen in melanoma are consistent with pyrimidine dimers (The Cancer Genome Atlas 2015). These patterns can be readily detected in tumors using standard NGS techniques because of the clonal nature of tumor formation. Mutations present early in neoplastic transformation are propagated to descendent tumor cells, where they are easily identified as well above the background error rate of sequencing (Fig. 4A). This is in contrast to early genotoxin‐associated mutations in normal tissues, which are present in only a minority of cells among a larger unmutated population, and where far more sensitive methods are required.

The primary challenge in performing this type of spectral analysis has been that somatic tumor mutations are the result of the cumulative mutational processes incurred by the founding cancer cell's lineage since embryogenesis. As such, it is necessary to deconvolute the relative contributions of each of these mutational processes. Alexandrov et al. were the first to report the use of nonnegative matrix factorization, a statistical method developed for decomposition of multivariant data, to computationally parse out constituent mutational processes based on both the specific mutation type (ie, G→T/C→A) and the identity of the adjacent 5′ and 3′ bases (Alexandrov et al. 2013). In their initial work, the authors reported 21 “mutational signatures” (or “trinucleotide signatures”) across the TGCA data set, with some of the signatures exhibiting high tumor‐type specificity (Alexandrov et al. 2013). Recent analysis of tumor sequencing data, comprising 4645 whole genomes and 19,184 exomes, has validated the vast majority of the initially reported signatures, as well as further expanded the number of mathematically defined signatures to now include a total of 49 single‐base substitution signatures, 11 doublet‐base substitution signatures, 4 clustered‐base substitution signatures, and 17 small insertion/deletion signatures (Alexandrov et al. 2018).

Mutational signatures have risen to prominence in the genomic literature over the last 5 years (reviewed in (Phillips 2018)), but they are not without limitations. Signatures are computationally derived. Some portion of the described signatures could be computational artifacts or subfeatures within other processes. Furthermore, the bulk of research on mutational signatures has focused on their presence in tumors, for the practical reasons described above. The signatures observed in a tumor may not fully recapitulate processes in normal tissues. Signatures in tumors arise from both endogenous and exogenous sources (Alexandrov et al. 2013; Alexandrov et al. 2015; Alexandrov et al. 2018) and are an amalgamation of mutagenic processes that may be somewhat biased by clonal sweeps that occur during tumor formation when effects unrelated to exposure‐associated mutagenesis are operative. Recent work using error‐corrected sequencing to study aflatoxin‐induced mutations in normal mouse tissue demonstrated the low‐frequency signature to be distinctly different from that observed in the tumor itself. This suggests additional mutagenic processes may have developed during tumorigenesis that were unrelated to aflatoxin exposure (Chawanthayatham et al. 2017; Fedeles et al. 2017).

For most genetic toxicologists, a forensic analysis of the mutational processes that led to clonal tumors is only useful insofar as the knowledge can be applied for prospectively screening new compounds. Sequencing human cancers that follow natural exposures, similar to sequencing of family trios to infer germ line processes that introduce mutations between generations (Fig. 4C), is simply not a practical tool in this regard. Most conventional genotoxicity assays are not equipped to take advantage of trinucleotide signature analysis due to their reliance on selective markers with a narrow nucleotide repertoire which can significantly bias observed spectrum (Fig. 4D). Simple, and even trinucleotide, mutational spectrums can be assessed from transgenic rodent assays by manually picking hundreds of phage plaques for sequencing, but in addition to being very labor intensive, the approach is still complicated by an incomplete repertoire of three base‐pair groups within the small reporter genes and the fact that synonymous mutations do not result in phenotypic changes.

A less biased approach for experimentally obtaining detailed mutational spectra without any biological selection is cloning of single cells after compound exposure followed by large‐scale sequencing (Fig. 4B). In an outstanding recent study by the Nik‐Zanal group, the authors carried out whole‐genome sequencing on induced pluripotent stem cells that were cloned from populations treated with nearly 80 known or suspected carcinogens, identifying dozens of distinct signatures (Kucab et al. 2019). This more than quadrupled the existing collection of signatures that have been experimentally ascribed to from exogenous sources—a list which will undoubtedly continue to grow (Chawanthayatham et al. 2017; Huang et al. 2017; Ng et al. 2017; Boot et al. 2018).

Cultured cells cannot fully recapitulate all the metabolic and distribution complexities of *in vivo* exposures and single‐cell cloning is not trivial (Blokzijl et al. 2016). However, the extensive signature knowledge and mathematical methods generated from both this approach and from genotyping tumors can be readily applied to the above‐described new sequencing technologies. Many of these have sufficient accuracy to detect low‐frequency genotoxin‐induced mutations without need for clonal expansion of any form (Fig. 4E). This opens the possibility of being able to assess mutational signatures in any cell type from any tissue from any species directly from extracted DNA (Chawanthayatham et al. 2017). Much remains to be done in this emerging space, but the future remains bright for its applications in genomic toxicology.

### “Neo‐Genotoxicity”: Genome Engineering Technologies

The classic fields of genetic toxicology and environmental mutagenesis have typically focused on the effects of broadly acting DNA damaging chemicals and their effects to human health. However, the emergence of new genetic manipulation technologies, what we term “neo‐genotoxins,” presents both new challenges and new opportunities for the field. A critical aspect of these tools, especially from a regulatory perspective, is determining their specificity in altering the genome in the desired way. Like traditional chemical mutagens, off‐target DNA cutting or gene mis‐insertion could increase the risk of cancer by inadvertently interrupting an oncogene or tumor suppressor. However, unlike randomly acting small molecules, the rules for predicting where in the genome this might happen, and the technical complexities for site‐specific screening, are completely different.

With the development of programmable endonucleases, such as zinc‐finger nucleases, transcription activator‐like effector nucleases, and, most recently, CRISPR/Cas nucleases, it is now possible to make targeted genomic alterations *in situ* (reviewed in (Gaj et al. 2013)). In theory, the 20–40 bases targeted by these enzymes should be more than sufficient to ensure complete specificity, but the presence of pseudogenes, human genetic variation, and a tolerance for sequence changes in the recognition sequence, can reduce site specificity (Lessard et al. 2017). *In silico* methods have been developed to help predict off‐target effects of these nucleases, especially for the CRISPR/Cas family of endonucleases, but have shown only moderate concordance with experimental data (reviewed in (Chuai et al. 2017)).

Using modern sequencing platforms, several unbiased methods have been developed to detect the presence of double‐strand breaks. A primary concern with these technologies is the hundreds to thousands of potential off‐target sites that exist across the genome. Further complicating the issue is that the probability of cutting off‐target sites can vary by several orders of magnitude which means that brute force sequencing may not be sensitive enough to detect rare off‐target events. While the specifics of each approach are different, they largely depend on using *in vitro* digestion with the nuclease in question followed by the introduction of a known universal sequence *via* ligation/integration or the cell's homologous recombination machinery that can be selected by PCR or targeted pulldown. These methods have reported a wide range of off‐target cutting depending on the method used (Fu et al. 2013; Frock et al. 2015; Tsai et al. 2015; Cameron et al. 2017; Tsai et al. 2017). There is a substantial need for more accurate and sensitive methods to detect off‐target cut sites.

A notable limitation of these methods is the inability to practically assess off‐target effects *in vivo*, which will be critical for regulatory testing and widespread medical use of genome‐editing technologies. To date, we are aware of only one *in vivo* method, termed “Verification of in vivo Off‐targets” (VIVO), that has been published. This approach uses a combination of *in vitro* off‐target detection with evaluating the observed off‐target sites seen in the *in vitro* data for characteristic deletion events caused by *in vivo* expression of CRISPR/Cas9 in mouse liver (Tsai et al. 2017; Akcakaya et al. 2018). Further complicating matters is that the highly sequence‐dependent nature of both on‐target and off‐target effects makes animals untenable surrogates for assessing genotoxicity induced by human‐genome targeted nucleases.

The clinical importance of neogenotoxins has become even more apparent with the emergence of cell‐based therapies. While cells do not constitute a genotoxin *per se*, the genetic engineering and potential for clonal selection of mutation‐harboring subpopulations during their development can lead to increased risk of acquiring cancer from within the transplanted cells. For example, recent studies have shown that genome editing using CRISPR‐Cas9 results in *TP53*‐mediated DNA damage response and cell‐cycle arrest. Consequently, there is a strong selective advantage for cells harboring inactivating mutations in this important tumor suppressor (Haapaniemi et al. 2018; Ihry et al. 2018; Sinha et al. 2018). In other words, the effect of even perfectly accurate on‐target cutting is natural selection of cells bearing the most common genetic driver in all human cancers. These issues, and others that have not yet been discovered, are likely to complicate therapeutic applications involving genetically engineered cells, such as for regenerative medicine or CAR‐T‐based cancer therapies. Technologies for accessing these risks will need to be extremely accurate, quickly adaptable to new targets, and equally applicable to *in vitro* preclinical usage as to *in vivo* human studies—a tall order by any estimation.

### Carcinogenicity vs. Mutagenicity

While essentially all human mutagens are carcinogens, the reverse is not always true. Mutagenesis is an imperfect surrogate for cancer risk. Nonmutagenic carcinogens may drive neoplasia through inflammation, epigenetic modifications, and endocrine disruption that drives aberrant cellular proliferation (Ohshima et al. 2003; Baccarelli and Bollati 2009; Soto and Sonnenschein 2010). In these cases, classic selection‐based mutagenesis assays would not easily detect these compounds as likely carcinogenic, indicating why 2‐year rodent studies remain a safety requirement for new drug approval.

A number of recent reports show that clonal expansion of cells harboring somatic mutations in cancer‐associated genes is a normal part of aging (reviewed in (Risques and Kennedy 2018)). Because non‐genotoxic carcinogens are generally believed to accelerate carcinogenesis by forcing unregulated cell division, clonal expansions of mutations could be used as a marker of emerging ability to proliferate outside the confines of the normal regulated tissue architecture (Salk and Horwitz 2010). The development of ultra‐accurate ecNGS may offer a way to quantify these expansions and correlate their presence with environmental exposure or potentially cancer risk. Approaches could involve the sequencing of large panels of cancer driver genes or hypermutable portions of the genome for clonal expansions. A similar idea has been used in studying somatic evolution in dysplastic and cancerous tissue (Salk et al. 2009; Naxerova et al. 2017; Baker et al. 2019). Detection of very early preneoplastic changes at the cellular level by observing accelerated growth of small clones could be carried out in conjunction with mutagenesis screening using the same ecNGS methods. For an in‐depth discussion on this topic, please see the accompanying review by Parsons and colleagues (Harris et al., 2019).

## FUTURE APPLICATIONS AND CONCLUSIONS

The utility of modern sequencing platforms has expanded well beyond the initial use of sequencing DNA for genome assembly and germ line variant detection, for which they were originally developed. While in its infancy, these technologies are ushering in a renaissance for the study of genotoxicity and somatic mutagenesis. The digital nature and massive scale at which these technologies operate is already providing rich data sets that are orders of magnitude beyond that which was available to the field's pioneers.

Ultimately, the technologies and methods that we have described here will be deployable for direct monitoring of exposures in human populations—a concept famously envisioned by William Thilly more than three decades ago (Sattaur 1985). Widely recognized environmental carcinogens such as aflatoxin and aristocholic acid cause thousands of cancer deaths globally per year, but, at the current time, it is impossible to know which individuals may have been exposed during their lives and are at the greatest risk (Ng et al. 2017). From the point of view of an individual, routine screening in at‐risk populations could identify those who would most benefit from close clinical surveillance.

From a public health perspective, population testing could aid in identifying regional exposure hot spots where source control efforts could be most effective. Numerous statistically defined “cancer clusters” have been described, frequently near industrial sites (Thun and Sinks 2004). New tools that more directly link chemical exposure of individuals to an instance of cancer could empower communities with objective data to more effectively demand cleanup and provide local governments and regulators with early detection tools to prevent clusters in the first place.

**Figure 5:**
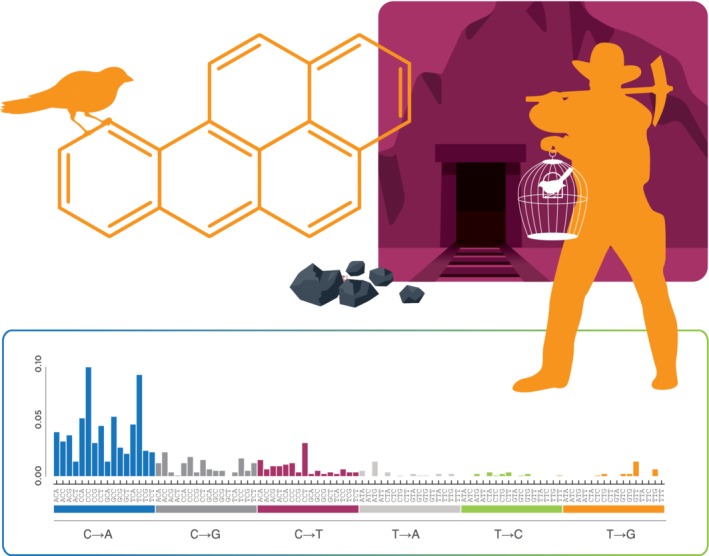
Canary‐in‐a‐coal‐mine: a century later. A hundred years ago, at the suggestion of John Scott Haldane, caged canaries were routinely brought into British coal mines as an early warning sign of human‐relevant toxic gases. Although their routine use ceased in the 1980s, the broader concept of using sentinel species to infer the presence of environmental hazards remains highly germane in modern genetic toxicology. Should it have been possible to collect and analyze a DNA sample from one of Haldane's birds using modern ecNGS techniques, it is quite likely that the mutagenic signature of benzo[a]pyrene could have been identified and used to inform efforts to mitigate the environmental cancer risk. Other naturally present sentinel organisms, including humans themselves, can be similarly used.

Due to the generalizability of NGS technologies to any source of DNA, surveying native organisms for mutagenic signatures in their genome would allow for environmental monitoring for the presence of mutagens. An amusing, yet entirely appropriate, analogy is the proverbial canary‐in‐a‐coal mine; in this modern rendition, it is the canary's genome that serves as a biosensor for mutagenic coal dust (Fig. 5). We envision that many of the varieties and applications of the new technologies outlined in this review can be combined to obtain a more complete picture of genotoxicity and cancer risk both in model systems and humans. The use‐cases described herein are likely to be only the beginning of our needs as we look toward engaging with mutagenic new environments, such as interplanetary space, and consider new high‐risk medical frontiers, such as gene editing of the germ line. The full breadth of applications for these new tools remains to be seen, but their use will undoubtedly offer new avenues of research and further drive development of technologies that will carry us through the next 50 years.

## AUTHOR CONTRIBUTIONS

S.R.K. and J.J.S. conceptualized the review topics. S.R.K. wrote the initial manuscript draft. S.R.K. and J.J.S. contributed to the figures and manuscript.

## Conflict of Interest

J.J.S. is an employee and equity holder at TwinStrand Biosciences. S.R.K. is a paid consultant and equity holder at TwinStrand Biosciences and a paid consultant for Wilcox & Savage, PC.
